# Phosphorus and base cations drive contrasting root dynamics in a central Amazon forest

**DOI:** 10.1007/s11104-026-08303-2

**Published:** 2026-02-02

**Authors:** Jéssica Schmeisk-Rosa, Kelly M. Andersen, Amanda L. Cordeiro, Anna Carolina Martins Moraes, Ana Cláudia Francisco Salomão, Rafael Leandro de Assis, Raffaello Di Ponzio, Renata Vilar de Almeida, Maria Pires Martins, Hellen Fernanda Viana Cunha, Nathielly Pires Martins, Sheila Trierveiler de Souza, Gyovanni Augusto Aguiar Ribeiro, José Augusto Salim, Érick Oblitas, Sara Deambrozi Coelho, Adriana C. Conceição, Bruno Takeshi Tanaka Portela, Oscar J. Valverde-Barrantes, José Luís C. Camargo, Patrick Meir, Anja Rammig, Iain P. Hartley, Carlos Alberto Nobre Quesada, Laynara F. Lugli

**Affiliations:** 1 Coordination of Environmental Dynamics, National Institute for Amazonian Research, Manaus, Brazil; 2 Faculty of Environment, Science and Economy, University of Exeter, Exeter, UK; 3 Latin America Department, Missouri Botanical Garden, Saint Louis, MO USA; 4 Department of Plant & Microbial Biology, University of Minnesota, St Paul, MN USA; 5 Biological Dynamics of Forest Fragment Project, National Institute for Amazonian Research, Manaus, Brazil; 6 Vale Institute of Technology, Belém, Brazil; 7 Programa de Pós Graduação Em Ecologia, Conservação E Manejo da Vida Silvestre, Universidade Federal de Minas Gerais, Belo Horizonte, Brazil; 8 TUM School of Life Sciences, Technical University of Munich, Freising, Germany; 9 Plant Biology Department, Institute of Biology, State University of Campinas, São Paulo, Brazil; 10 International Centre of Tropical Biodiversity, Department of Biological Sciences, Florida International University, Miami, USA; 11 School of Geosciences, University of Edinburgh, Edinburgh, UK; 12 Research School of Biology, Australian National University, Australian Capital Territory, Canberra, Australia

**Keywords:** Tropical forest, Nutrient availability, Nutrient fertilization experiment, Fine root stock, Fine root turnover

## Abstract

**Background and aims:**

In highly weathered soils of central Amazonia, where nutrients such as phosphorus (P) and base cations are scarce, fertilization experiments have demonstrated above- and belowground effects on total net primary productivity (NPP). This study examined how fine root stocks and turnover responded to added nutrients over a two-year period. We predicted that adding a limiting nutrient would decrease fine root stocks and increase turnover, with the strongest effects from P, followed by base cations, and no response to N.

**Methods:**

Fine roots (< 2 mm diameter) were sampled from the 0–30 cm soil layer in a low-fertility primary forest in central Amazon subjected to a large-scale factorial experiment adding P, base cations, and N over two years. Fine root turnover was calculated as the ratio between fine root productivity, measured with in-growth cores, and fine root stock.

**Results:**

Fine root stocks remained unchanged with nutrient addition. However, P increased root turnover by 23% and 48% in the first and second years, respectively, while base cations addition reduced turnover by 24% in year two. N had no significant effect, though a trend toward reduced turnover was observed in the second year.

**Conclusion:**

The results of this study show that fine root standing stock and turnover in the central Amazon are regulated by soil nutrient availability, especially P and base cations. The contrasting responses observed suggest distinct belowground resource-use strategies for different nutrients, shaped by the nutrient specific mobility in the soil and physiological role in the plant.

**Supplementary Information:**

The online version contains supplementary material available at 10.1007/s11104-026-08303-2.

## Introduction

Soil nutrient availability is a key factor for net primary productivity (NPP) in forest ecosystems, especially in tropical forests (Vitousek et al. 2010). The effects of nutrient availability on forest functioning vary depending on the nutrient and ecosystem components, reflecting the complexity of these interactions (Graefe et al. 2010; Homeier et al. 2012; Alvarez-Clare et al. 2013; Manu et al. 2022; Cunha et al. 2022; Wright and Harms 2024). On top of that, interactions between soil conditions and climatic variability across the basin play a crucial role in species distribution and forest dynamics (Malhi et al. 2009; Aragão et al. 2009; Mercado et al. 2011; Quesada et al. 2012). Approximately 60% of the Amazon basin is composed of low-fertility soils (Quesada et al. 2010, 2012). Large parts of the Amazon region are established on highly weathered soils, where the low availability of rock-derived phosphorus (P) and the base cations potassium (K), calcium (Ca), and magnesium (Mg), potentially impact the growth of forests (Quesada et al. 2010, 2012; Quesada and Lloyd 2016). In contrast, soil nitrogen (N) accumulates over time through biological fixation and atmospheric deposition (Hedin et al. 2003), resulting in relatively high levels of N in highly weathered soils of central and eastern Amazon (Quesada et al. 2010).

The net primary productivity (NPP) allocation to fine roots (roots < 2 mm) represents between 5 and 49% of total lowland tropical forests NPP (Huaraca Huasco et al. 2021). Fine roots are critical for acquiring nutrients and water, but also for supporting the overall function and resilience of the root system under environmental stress (Freschet et al. 2021). Understanding root dynamics is, therefore, essential to gain insights into ecosystem functioning and responses to future global changes. For instance, in the Amazon basin, low soil fertility is related to lower belowground productivity but greater root biomass storage compared to high soil fertility (Aragão et al. 2009; Quesada et al. 2012). To thrive in the nutrient-limited conditions of Amazonian soils, plant species have evolved strategies that allow them to sustain productivity and support nutrient, including mycorrhizal symbioses, organic acid exudation, acid phosphatase exudation, and intensive fine root foraging (Lugli et al. 2020; Martins et al. 2021; Reichert et al. 2022).

The equilibrium between root production, biomass accumulation, and replacement of older roots to optimise resource acquisition affects ecosystem function and dynamics. Fine root dynamics can be described using three complementary metrics. Fine root productivity represents plant investment in belowground growth for resource acquisition over a given period; fine root stock represents the current standing biomass of fine roots at a given point in time, integrating past production and mortality; and fine root turnover describes the rate at which root biomass is replaced by new growth and is inversely related to root lifespan (Yavitt et al. 2011; Huaraca Huasco et al. 2021). The variability of these three parameters directly influences the adaptability and resilience of forest ecosystems to varying environmental conditions and highlights the complex interactions between environmental properties and plant morphological and physiological characteristics (Vogt et al. 1996; Huaraca Huasco et al. 2021; Freschet et al. 2021; Cusack et al. 2021). In resource-poor environments, such as in vast areas of the Amazon basin, conservative mechanisms are common to maximise nutrient use, resulting in slower C dynamics when compared to more fertile regions (Bloom et al. 1985; Yavitt et al. 2011; Lugli et al. 2021; Reichert et al. 2022). Therefore, maintaining fine root biomass standing stock by reducing root turnover to prevent nutrient loss is prioritised over the productivity of new roots in places with low nutrient availability (Huaraca Huasco et al. 2021). Conversely, in nutrient-rich environments, C cycling in roots tends to be faster, shifting C allocation investments from long-lived root biomass stocks to increased productivity and turnover of fine roots, leading to more acquisitive plant economic strategies (Aragão et al. 2009; Huaraca Huasco et al. 2021; Cusack et al. 2021). As a consequence of these dynamics, the fate of root C significantly impacts the C soil pool and the dynamics of the forest (Poirier et al. 2018; Sokol and Bradford 2019; Cunha et al. 2022; Kengdo et al. 2023).

Nutrient manipulation experiments are essential for understanding the effects of nutrient limitations on ecosystem dynamics and resilience (Hofhansl et al. 2016). Experimental studies in other tropical forests have investigated the response of fine root dynamics to nutrient additions. In a seasonal lowland tropical forest in Panama growing in relatively fertile soils, fine root stock decreased after four years of K addition and fine root turnover increased (Yavitt et al. 2011). After 14 years of fertilisation, combined addition of N, P, and K resulted in a 50% reduction in fine root stocks in the 0–10 cm soil layer (Wurzburger and Wright 2015). In a humid semi-deciduous tropical forest in Uganda, also growing on relatively fertile soils, fine root stocks decreased with N and K addition, fine root production decreased with K addition and turnover was not affected in the 0–30 cm deep layer after two years of fertilization (Manu et al. 2024). These findings highlight the importance of multiple nutrients for root function across the tropical forests (Manu et al. 2024), and the need to better understand how different nutrients and their interactions regulate fine root dynamics. To date, most of the available evidence comes from relatively fertile tropical forests and focuses primarily on fine root stocks and productivity, with less emphasis on fine root longevity and turnover, especially under low nutrient availability (Yavitt et al.^2011^; Wurzburguer and Wright 2015; Lugli et al 2021; Manu et al 2024). However, considering fine root stocks, productivity, and especially turnover, it becomes possible to assess not only how much C is allocated to the root system, but also how rapidly this C is renewed and returned to the soil (Freschet et al. 2021; Cusack et al. 2021). This integrated perspective allows us to distinguish short-term responses from more persistent adjustments in belowground functioning. Additionally, it provides a more robust basis for evaluating how highly nutrient-poor *terra firme* forests in central Amazonia may respond to sustained changes in nutrient availability under ongoing global environmental change.

The current study builds on the findings of the Amazon Fertilisation Experiment (AFEX) (Lugli et al. 2021; Cunha et al. 2022) by incorporating fine root productivity data and introducing new insights into fine root stocks and turnover, ultimately exploring the influence of phosphorus (P), base cations, and nitrogen (N) on overall root dynamics. The Amazon Fertilisation Experiment (AFEX), conducted in a primary low-fertility *terra firme* forest in central Amazonia, has shown that the addition of P increased fine root productivity in the 0–30 cm soil layer across two years, whereas base cation additions increased fine root productivity in the first year of additions (Lugli et al. 2021). After two years, only P maintained significant effects, enhancing fine root and canopy NPP without impacting stem growth (Cunha et al. 2022). Thus, fine root productivity can be strongly affected by changes in soil resources, particularly in locations where nutrient availability is low (Wurzburger and Wright 2015; Lugli et al. 2021). These findings indicate a faster C cycle, resembling naturally fertile forests like those from the western Amazon (Aragão et al. 2009; Quesada et al. 2012). Furthermore, the observed stronger responses of P addition after two years suggest that C dynamics have yet to reach a new equilibrium, with potential future impacts on ecosystem functioning and on C balance.

Fine root productivity, standing biomass stock, and turnover represent the pathways of C allocation within root systems, which are essential for determining belowground and whole-ecosystem C dynamics (Freschet et al. 2021; Cusack et al. 2021). Thus, we expect reduced root standing stock biomass and increased root turnover with the addition of the limiting nutrients. Specifically, given that rock-derived nutrients such as P and base cations are more limiting than N in central Amazonian forests, we predict the following responses to nutrient additions: (i) a reduction in plant community fine root stocks and a more pronounced increase in overall root turnover with P addition, driven by the strong productivity gains associated with enhanced P availability; (ii) similar but less pronounced effects with cation addition, reflecting their low natural availability as rock-derived nutrients; (iii) more substantial effects in the second year of nutrient addition than in the first, suggesting cumulative impacts over time; and (iv) no significant changes in root dynamics with N addition, given its relatively high availability in these ecosystems.

## Material and methods

### Site description

The study site is located in a continuous old-growth evergreen forest growing in the plateaus of the ZF-3 Reserve, AM, Brazil (02° 25′ 00'' S; 59° 43′ 00' W), managed by the Biological Dynamics of Forest Fragments Project (BDFFP) (Laurance et al. 2018), a collaborative project between the National Institute for Amazonian Research (INPA) and the Smithsonian Tropical Research Institute (STRI). The forests are classified as Lowland Dense Ombrophilous Terra Firme forests (Santos 2014), with a mean air temperature of 26 °C and mean annual precipitation of 2400 mm, and a dry season from July to October (Tanaka et al. 2014). The soil types are Ferralsols and Acrisols (World Reference Base for Soil Resources; or Oxisols and Ultisols in the U.S. Soil Taxonomy (Quesada et al. 2010, 2011), which are highly weathered and nutrient-depleted, with particularly low concentrations of rock-derived elements (mean total P 85.7 mg kg ^−1^, pH 4.2, total N 0.19% and sum of total bases 0.22 cmol_c_ kg^−1^ for the 0–30 cm (Ribeiro, 2023). The research site has high tree species diversity, with about 280 species (> = 10 cm DBH) per hectare (Cunha et al. 2022). For a more detailed description of the site, see Lugli et al., (2021) and Cunha et al., (2022).

The AFEX experiment consists of 32 plots distributed in 4 blocks, with at least 200 m distance between them, installed in plateau areas with similar soil, vegetation, and terrain. In each block, there are eight 50 m × 50 m plots (at least 50 m apart from each other), with one control plot and seven plots with nutrient addition treatments: P, N, CAT (Ca, Mg, K), P + N, P + CAT, N + CAT, P + N + CAT, resulting in 4 plots per treatment (n = 4). Nutrient additions were split annually into three equal applications throughout each wet season, with the first application occurring between May and June 2017, at the following total rates: (1) N: 125 kg ha^−1^ yr^−1^ as urea; (2) P: 50 kg ha^−1^ yr^−1^ as triple superphosphate, and (3) base cations: 50 kg ha^−1^ yr^−1^ for Ca, 20 kg ha^−1^ yr^−1^ as Mg in the form of dolomitic limestone, plus 50 kg ha^−1^ yr^−1^ as potassium chloride for K. Dry fertilisers were added to the soil surface by hand, covering the plot area, including the surface of the ingrowth cores (Lugli et al. 2021). Roots in this study were sampled in the center 30 m x 30 m (900 m^2^) of each plot at the community level, with all fine roots collected regardless of species identity or growth form (woody vs. non-woody), and our results represent the response of root dynamics for the first two years of nutrient addition from 2017 to 2019.

### Fine root collections

Fine root stock was sampled in August 2017 and September 2019. The August 2017 sampling represents the 2017/2018 period (year 1) and the September 2019 sampling represents the 2018/2019 period (year 2) for fine root stock. No stock measurements were conducted in 2018. In both fine root stock campaigns, soil cores were collected from previously undisturbed locations within the plot, avoiding any areas affected by prior sampling. Four ingrowth core campaigns (collections every three months) were used to determine the annual productivity. Campaigns from August 2017 to September 2018 were used for year 1 and campaigns from September 2018 to September 2019 were used for year 2.

In both fine root stocks and fine root productivity sampling campaigns, five soil cores per plot were collected. Cores measured 12 cm in diameter and 30 cm in depth. In the field, each of the five cores was separated by depth and homogenised to produce two subsamples per plot (0–10 cm and 10–30 cm; n = 64). All fine roots from each soil depth subsample were sampled using a modified method introduced by Metcalfe et al. 2007. Briefly, fine roots were manually extracted from the soil in four intervals of 15 min and curves were fitted to estimate total root biomass over 180 min (see details in Metcalfe et al. 2007 and Lugli et al. 2021). The Michaelis–Menten asymptotic curve (Eq. 1) was the best model to extrapolate to the amount of root biomass that would be sampled over 180 min:

$$y=\frac{\alpha \times x}{\beta \times x }$$

where $y$ is the total fine root biomass estimated in each sample after 180 min of sampling, $x$ is accumulated time (15 to 180 min), $\alpha$ and $\beta$ fitted parameter from the equation for each plot and depth.

In the lab, fine roots were separated into diameter classes < 2 mm and ≥ 2 mm, cleaned and dried at 60 ºC until constant mass and weighed. For our calculations, we used fine roots < 2 mm in diameter. Fine root stocks were calculated as the total dry mass of fine roots per hectare per soil depth (0–10 cm and 10–30 cm) and expressed in Mg C ha^−1^. In this study, the mean C fraction in root dry mass was considered equal to 0.439 (Lugli et al. 2021).

Fine root productivity from Lugli et al., (2021) and Cunha et al., (2022) was used in this study (Table S1 and S2—Online Resource). To estimate fine root productivity, ingrowth cores (2 cm plastic mesh) 30 cm deep and 12 cm in diameter were installed in August 2017 following Metcalfe et al., (2007). Ingrowth cores were installed in the same cores where roots were sampled for fine root stock analysis (five cores per plot, n = 32), and root-free soils from each plot and respective depth (0–10 cm and 10–30 cm; n = 64) were inserted into each ingrowth core. For each core, soil from each depth layer was gently repacked by hand to approximate the in-situ structure and bulk density and to avoid large air gaps or excessive compaction. Ingrowth cores were collected every three months after installation. Fine root productivity was calculated as the dry mass of roots produced per hectare by depth (0–10 cm and 10–30 cm) for a year, expressed in Mg C ha^−1^ yr^−1.^

Fine root turnover was obtained by the ratio between annual fine root productivity and fine root stocks per plot from the 0–30 cm soil depth (n = 64, Freschet et al. 2021). Fine root turnover for year 1 was obtained by dividing annual fine root productivity (from ingrowth cores collected between August 2017 and September 2018) by the fine root stock measured in August 2017; similarly, fine root turnover for year 2 was obtained by dividing annual fine root productivity (from ingrowth cores collected between September 2018 and September 2019) by the fine root stock measured in September 2019. Fine root turnover was expressed in year^−1^, representing the rate at which root biomass is replaced annually, with higher values indicating faster turnover, meaning a shorter lifespan (Freschet et al. 2021). Since fine root turnover is mathematically the inverse of longevity or root lifespan (Freschet et al. 2021), turnover values were occasionally converted to fine root longevity (in days) to improve interpretability and facilitate comparisons across findings.

### Data analysis

Linear mixed effects models (LME) were used to evaluate the effects of each nutrient and all interaction terms (P × base cations × N) with our response variables: fine root stock and turnover (n = 32 plots) for the 0–30 cm depth. The analysis initially employed full factorial models to assess all potential interactions and the responses across years (Eq. 2). In the full factorial models, plots were incorporated as random factors, and years were analysed as both fixed and random factors to account for temporal variability. Simple factorial models were also used to observe the responses of the variables within each year (Eq. 3). In both models, nutrient presence or absence was included as fixed factors and experimental blocks were treated as random factors.

2 $$\text{Response variable}\sim \mathrm{P}*\text{base cations}*\mathrm{N}*\mathrm{Year}+(1|\mathrm{Year})+(1|\mathrm{Block}/\mathrm{Plot})$$

3 $$\text{Response variable}\sim \mathrm{P}*\text{base cations}*\mathrm{N}+(1|\mathrm{Block})$$

Full factorial models were simplified using backward elimination with the "step" function in the lme4 package (Bates et al. 2015), maintaining the random effects during the simplification process. The simplified models were extracted using the "get_model" function from the ‘lmerTest ‘ package (Kuznetsova et al. 2017), which reruns the final model to include only the fixed and random effects remaining after simplification. Effects of nutrient addition were considered statistically significant at p < 0.05.

For any simplified models with significant interactions, we conducted post-hoc analyses using the “emmeans” function in the ‘emmeans ‘ package (Lenth 2017). Pairwise comparisons of estimated marginal means were performed using the Tukey method (adjust = “tukey”) to determine differences in the mean values of the interacting variables. All models were conducted using ‘LMERConvienceFuncions ‘, the lmer() in the ‘lme4’, and ‘lmerTest’ packages (Bates et al. 2015; Kuznetsova et al. 2017; Tremblay and Ransijn 2020) and model fits were determined using the “check_model” function in the package ‘performance’(Lüdecke et al. 2021).

For the graphical representation of the effect of specific nutrients, all plots where a nutrient was not added (e.g., -P; n = 16 for one year) were compared with all plots where that nutrient was added (e.g., + P; n = 16) (Wright et al. 2011; Lugli et al. 2021). Similarly, for interactions in nutrient effects, results were compared across all plots where nutrients were added together and separately (e.g., -P-CAT, n = 8; -P + CAT, n = 8; + P-CAT, n = 8; + P + CAT, n = 8). All statistical analyses were run in the statistical program R version 4.1.1 (R Core Team 2024).

## Results

### Fine root dynamics with P addition

**Fig. 1:**
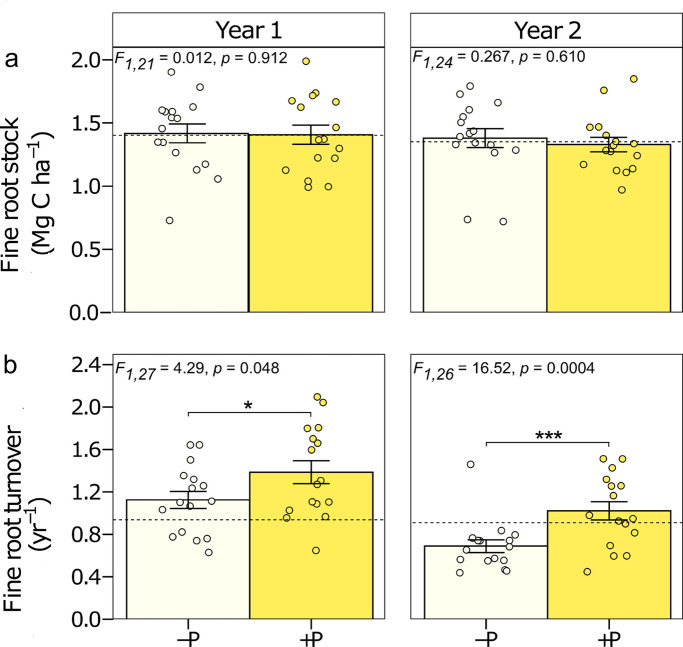
Fine root dynamics for the 0–30 cm soil depth for two years, with or without P-addition, in a lowland tropical forest in the central Amazon, Brazil. a. Fine root stock (Mg C ha^−1^) and b. Fine root turnover (yr.^−1^). Year 1 panel and Year 2 panel compare16 plots without or with P-addition (-P and + P respectively). Means ± 1SE are presented. Significance levels indicate differences among nutrient addition treatments within each year, as follows: *** for *p* < 0.001, ** for *p* < 0.01, and * for *p* < 0.05, respectively. The dotted lines represent mean values for the control plots (no nutrients added; *n* = 4 plots). To calculate the fine root turnover for Year 1, we used the fine root productivity values reported by Lugli et al., (2021), and for Year 2, we used the productivity values reported by Cunha et al. (2022)

No significant effect of P addition was observed on fine root stock in the top 30 cm of the soil (Fig. 1a). Fine root stock in the first year was 1.42 ± 0.07 Mg C ha^−1^ in -P plots and 1.41 ± 0.05 Mg C ha^−1^ in + P plots, and in the second year, 1.38 ± 0.07 Mg C ha^−1^ in -P plots and 1.33 ± 0.06 Mg C ha^−1^ in + P plots.

However, we found a significant increase in root turnover with added P both in year 1 and year 2 (Fig. 1b). In the first year of nutrient addition, fine root turnover was 23% higher in plots with P addition than in plots without P addition (-P: 1.12 ± 0.08 yr^−1^; + P: 1.38 ± 0.12 yr^−1^; F_1,27_ = 4.29, p = 0.048; Fig. 1b). The increase in root turnover translates to a decrease in root lifespan of approximately 60 days with P addition. In the second year, root turnover increased by 48% with + P when compared to -P plots (-P: 0.69 ± 0.06 yr^−1^; + P: 1.02 ± 0.08 yr^−1^; F_1,26_ = 16.52, p < 0.001; Fig. 1b), decreasing root lifespan by approximately 170 days. There were no significant differences between the mean fine root stock (F_1,24_ = 0.10, p = 0.75) or fine root turnover (F_1,28_ = 1.71, p = 0.20) indicating that mean values did not differ between year 1 and year 2 across P addition treatments. Descriptive statistics (mean ± SE) and ANOVA results for all depth intervals, years, and nutrients are provided in the Online Resource (Tables S1–S8).

### Fine root dynamic with base cations addition

**Fig. 2:**
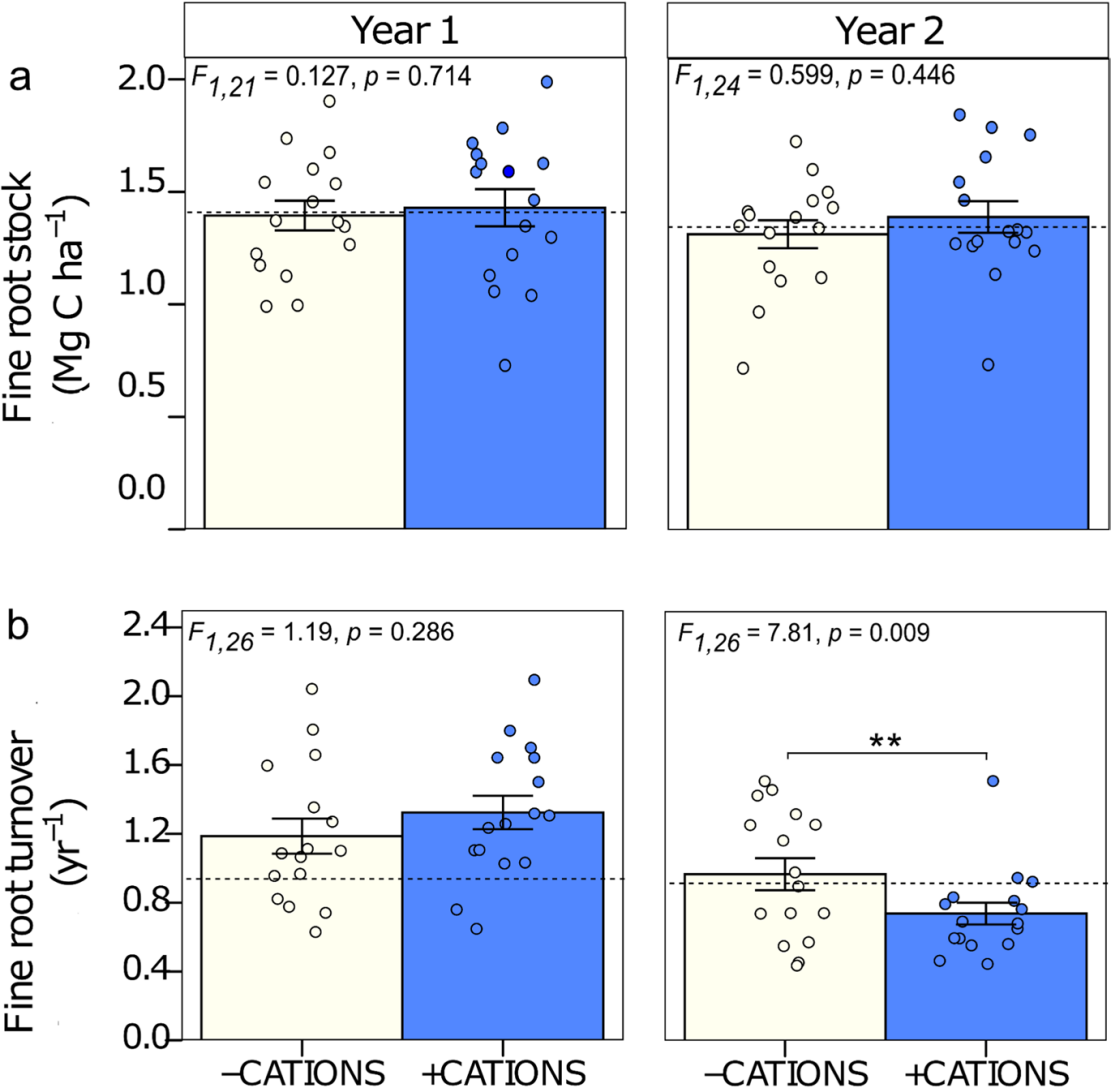
Fine root dynamic for the 0–30 cm soil depth for two years, with or without base cations-addition, in a lowland tropical forest in the central Amazon, Brazil. a. Fine root stock (Mg C ha^−1^) and b. Fine root turnover (yr.^−1^). Year 1 panel and Year 2 panel compare 16 plots without or with base cations-addition (-CATIONS and + CATIONS respectively). Means ± 1SE are presented. Significance levels indicate differences among nutrient addition treatments within each year, as follows: *** for *p* < 0.001, ** for *p* < 0.01, and * for *p* < 0.05, respectively. The dotted lines represent mean values for the control plots (no nutrients added; *n* = 4 plots). To calculate the fine root turnover for Year 1, we used the fine root productivity values reported by Lugli et al., (2021), and for Year 2, we used the productivity values reported by Cunha et al. (2022)

No significant effect of base cations addition was found on fine root stock in years 1 or 2 (Fig. 2a). Fine root stock in the first year was 1.39 ± 0.06 Mg C ha^−1^ in -CAT plots and 1.43 ± 0.08 Mg C ha^−1^ in + CAT plots (F_1,21_ = 0.127, p = 0.714) and in the second year it was 1.33 ± 0.06 Mg C ha^−1^ in -CAT plots and 1.39 ± 0.07 Mg C ha^−1^ in + CAT plots (F_1,24_ = 0.599, p = 0.446). No significant variation in fine root stock was detected between the years (F_1,24_ = 0.10, p = 0.75).

**Fig. 3:**
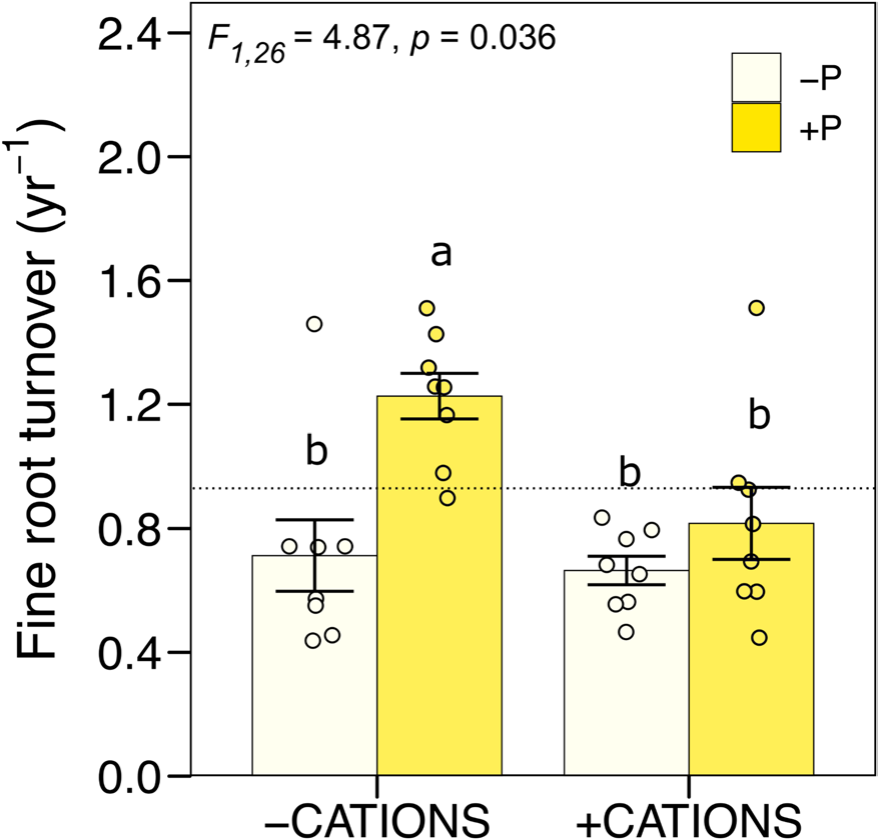
Fine root turnover (yr^−1^) for the 0–30 cm soil depth for year 2 in a lowland tropical forest in the central Amazon, Brazil. Fine root turnover (yr.^−1^) interaction between base cations and P. Each bar compares 8 plots without or with nutrient addition. Means ± 1SE (*n* = 8) are presented. The letters 'a' and 'b' indicate significant differences between groups (*p* < 0.05) according to Tukey's test. Groups sharing the same letter do not differ significantly. Dotted lines represent mean values for the control plots (no nutrients added; *n* = 4 plots)

There was a significant decrease in fine root turnover with base cation additions in year 2 only (Fig. 2B). Correspondingly, there was a significant interaction between CAT * Year (F_1,28_ = 8.05, p = 0.0084), with no differences between with and without base cation additions detected in Year 1 (F_1,26_ = 1.19, p = 0.286) and a 24% decline in fine root turnover in response to base cations additions in year 2 (-CAT: 0.97 ± 0.09 yr^−1^; + CAT: 0.74 ± 0.06 yr^−1^; F_1,26_ = 7.81, p = 0.0096; Fig. 2b). There was also a significant interaction between base cations and P in the second year, in which the effect of faster turnover with the addition of P was counteracted by addition of base cations (-CAT + P: 1.23 ± 0.07 yr^−1^; + CAT + P: 0.82 ± 0.12 yr^−1^; F_1,26_ = 4.87, p = 0.036; Fig. 3). Root lifespan was approximately 296 days when P was added without base cations. However, adding base cations alongside P cancelled this effect, reducing the effect of P on root turnover by 33% and extending root lifespan to 445 days.

### Fine root dynamic with N addition

**Fig. 4:**
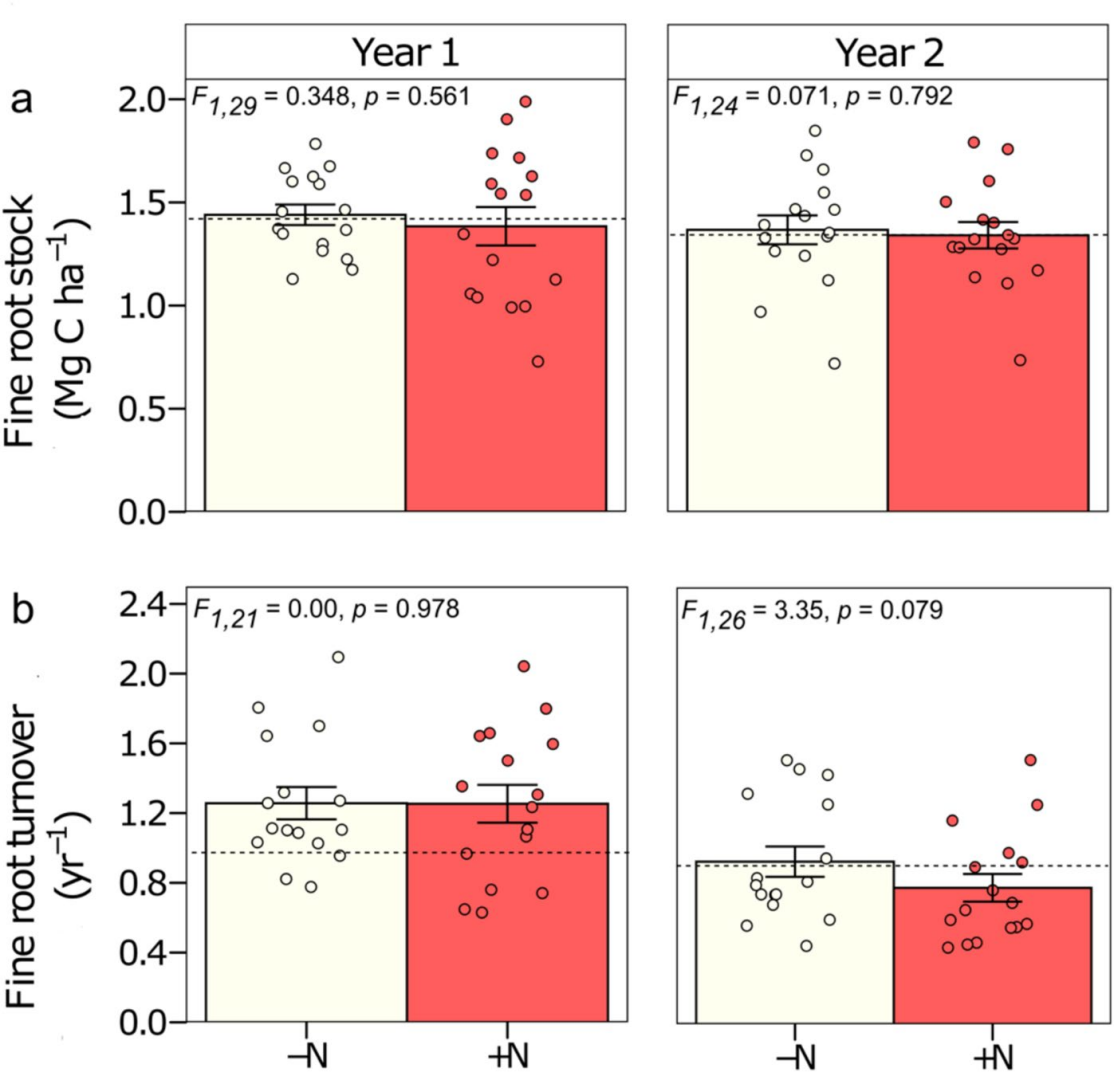
Fine root dynamic for the 0–30 cm soil depth for two years, with or without N-addition, in a lowland tropical forest in the central Amazon, Brazil. a. Fine root stock (Mg C ha^−1^) and b. Fine root turnover (yr.^−1^). Year 1 panel and Year 2 panel compare 16 plots without or with N-addition (-N and + N respectively). Means ± 1SE are presented. Significance levels indicate differences among nutrient addition treatments within each year, as follows: *** for *p* < 0.001, ** for *p* < 0.01, and * for *p* < 0.05, respectively. The dotted lines represent mean values for the control plots (no nutrients added; n = 4 plots). To calculate the fine root turnover for Year 1, we used the fine root productivity values reported by Lugli et al., (2021), and for Year 2, we used the productivity values reported by Cunha et al. (2022)

**Fig. 5:**
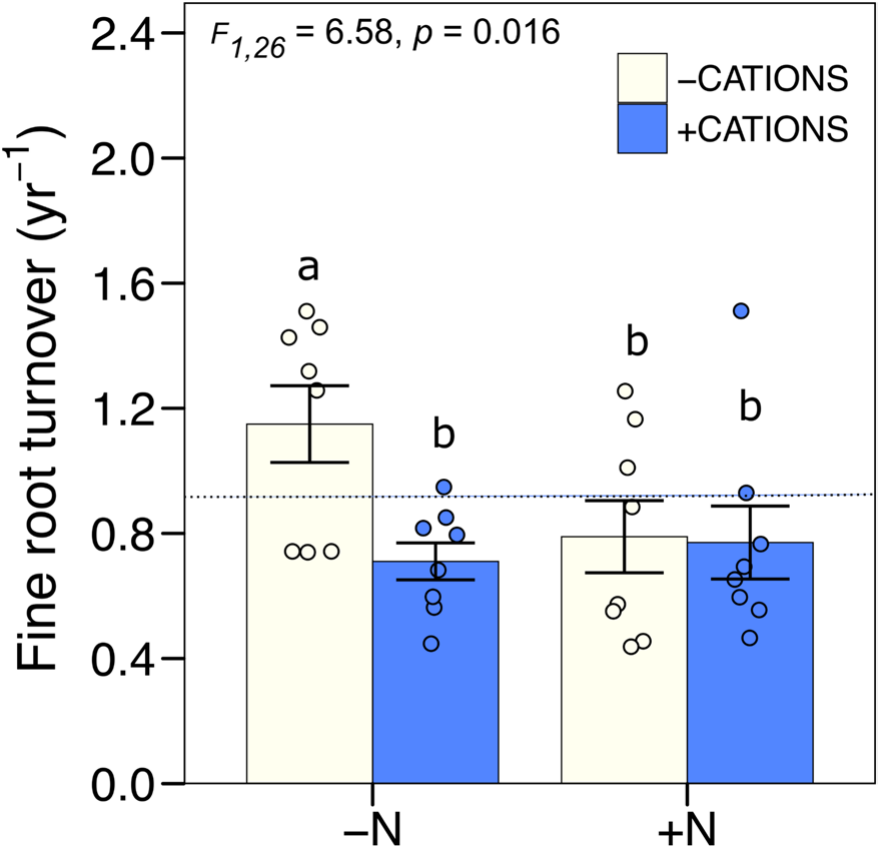
Fine root turnover (yr^−1^) for the 0–30 cm soil depth for year 2 in a lowland tropical forest in the central Amazon, Brazil. Fine root turnover (yr.^−1^) interaction between N and cation. Each bar compares 8 plots without or with nutrient addition. Means ± 1SE (*n* = 8) are presented. The letters 'a' and 'b' indicate significant differences between groups (*p* < 0.05) according to Tukey's test. Groups sharing the same letter do not differ significantly. Dotted lines represent mean values for the control plots (no nutrients added; *n* = 4 plots)

No significant effect was observed on fine root stock or in fine root turnover with N addition for years 1 and 2 (Fig. 4). However, the root turnover under N addition was slightly lower than without N addition in year 2 (F_1,26_ = 3.35, p = 0.079). Fine root stock in the first year was 1.44 ± 0.05 Mg C ha^−1^ in -N plots and 1.38 ± 0.09 Mg C ha^−1^ in + N plots (F_1,29_ = 0.348, p = 0.561) and in the second year, 1.37 ± 0.07 Mg C ha^−1^ in -N plots and 1.34 ± 0.06 Mg C ha^−1^ in + N plots (F_1,24_ = 0.071, p = 0.792). Fine root turnover in the first year was 1.26 ± 0.09 yr^−1^ in -N plots and 1.25 ± 0.11 yr^−1^ in + N plots (F_1,21_ = 0.00, p = 0.978), while in the second year these values were 0.93 ± 0.08 yr^−1^ in -N plots and 0.78 ± 0.08 yr^−1^ in + N plots (F_1,26_ = 3.35, p = 0.079), corresponding to an increase of approximately 176 days in root lifespan under N addition. No significant differences were observed between the two years in fine root stock (F_1,24_ = 0.10, p = 0.75) and fine root turnover (F_1,28_ = 1.71, p = 0.20) with N addition. Despite this, there was a significant interaction between N and base cations in year 2. We observed that root turnover was higher without N and base cations compared to adding either or both N and base cations (-N-CAT: 1.15 yr^−1^ and + N-CAT: 0.79 yr^−1^; F_1,26_ = 6.58, p = 0.016; Fig. 5), resulting in an increase in fine root lifespan of 145 days when N was added.

## Discussion

In the first two years of the nutrient addition experiment, root dynamics were responsive to increases in nutrient availability, with P addition eliciting the strongest changes across two years. While fine root stocks remained unchanged in all treatments, P addition accelerated fine root dynamics via increases in turnover. Surprisingly, base cations alone or combined with other nutrients also affected fine root C dynamics, but in the opposite direction as our predictions, decreasing fine root turnover and interacting with the effects of different treatments. After only two years of experimental treatment, detecting such responses in a hyper-diverse and slow-growing forest demonstrates the complexity and the plasticity in root dynamics, which may significantly affect C stocks and fluxes in the central Amazon forest.

Previous AFEX results demonstrated that fine root productivity (0–30 cm depth) increased 23% with P addition in the first year of the experiment (Lugli et al. 2021) and 29% after two years of P addition, in addition to a significant 19% increase in canopy productivity (Cunha et al. 2022). Our findings provide new insights, indicating that despite the increased productivity observed in these studies, fine root stocks remained stable due to higher fine root turnover. These results show that even in the short term, increased soil resource availability in this forest leads to more productive above and belowground compartments, especially for more dynamic organs such as leaves and roots (no changes in stem productivity were detected; see Cunha et al. 2022). Such faster C cycling resembles above and belowground C dynamics of other naturally fertile forests, such as the ones growing in the western Amazon (Aragão et al. 2009; Quesada et al. 2012), suggesting that this low-fertility forest may temporarily shift to a more “fast-cycling” state driven by short-lived fine roots and leaf tissues, with direct consequences for belowground C and nutrient cycling.

### Fine root dynamics with P addition

We predicted that the addition of limiting nutrients, especially P, followed by base cations, would reduce the fine root stock (Bloom et al. 1985). However, contrary to our expectations, none of the nutrient addition treatments had this effect. This suggests that adding nutrients over two years could have been insufficient to alleviate the plant’s nutritional limitation, as root stocks remained unchanged. In tropical moist forests, fine root stocks (0–30 cm depth) ranged from 0.76 Mg C ha⁻1 in a primary lowland forest in Borneo to 21.86 Mg C ha⁻1 in an undisturbed terra firme forest in the eastern Brazilian Amazon (Aragão et al. 2009; Huaraca Huasco et al. 2021), encompassing sites that differ in soil type and fertility. Our value of 1.34 ± 0.11 Mg C ha⁻1 in control plots lies toward the lower end of this spectrum of variation (Aragão et al. 2009). Such natural low values of fine root stocks could also help explain the observed lack of downregulation after nutrient addition. Thus, instead of further reducing fine root stock, responses to nutrient addition may occur via changes in other root attributes, such as increased fine root turnover and enhanced mycorrhizal associations, which can more efficiently exploit nutrient pulses in the soil (Freschet et al. 2021; Reichert et al. 2021).

In support of our first hypothesis, fine root turnover significantly increased in plots with added P. The combination of unchanged root stocks and increased root productivity resulted in increased fine root turnover, with the average fine root lifespan decreasing from 401 to 304 days after two years of P-addition. A shorter root lifespan leads to a generally younger and potentially more metabolically active root cohort in P-fertilised plots than longer-lived roots in -P plots. Since P has low mobility in the soil (Cole and Heil 1981), extensive root foraging for uptake is required. These younger fine roots might be particularly efficient in absorbing soil water and nutrients (McCormack et al. 2015; Freschet et al. 2021). Thus, although such more active young roots might also have higher respiration rates (McCormack et al. 2015), the costs of building new roots might be lower than the costs of root maintenance (*e.g.* longer lifespan and/or higher stocks). As a result, trees increase their investments in fine root productivity to forage the soil in response to the abundance of P (Wurzburger and Wright 2015; Lugli et al. 2021; Cunha et al. 2022). If the responsiveness to P addition increases with time, this would indicate that C dynamics in this forest have yet to reach a new equilibrium, with potential future changes in response intensity affecting these forests' functioning and the whole C budget. Our fine root productivity estimates come from soil volumes previously sampled and refilled with root-free soil, as inherent to the ingrowth core method, which may differ slightly from undisturbed conditions. These methodological constraints and the relatively short duration of the experiment should be considered when interpreting absolute values, but do not undermine the robustness of our treatment comparisons or the clear patterns detected.

The increased fine root turnover with greater soil P availability could also be a response to changes in the nutritional quality of the root tissue (Lugli et al. 2021). Increasing tissue quality, for example through higher nutrient concentrations in fine roots, is expected to increase (i.e. accelerate) fine root decomposition rates, which ultimately influence and are influenced by the soil microbial community (Silver and Miya 2001). Additionally, faster fine root turnover could be a strategy to cope with increased root herbivory due to changes in root tissue stoichiometry (Wells et al. 2002; Freschet et al. 2021). With abundant resources (*e.g.*, P), trees might replace fine roots more frequently through higher turnover instead of investing in secondary compounds and greater tissue density to deter herbivores (Coley et al. 1985; Wells et al. 2002; Freschet et al. 2021). The short-lived fine roots increase the frequency of C pulses to the soil as a result of faster fine root dynamic, potentially changing soil C stocks (Rasse et al. 2005; Metcalfe et al. 2007; Poirier et al. 2018; Sokol and Bradford 2019; Kengdo et al. 2023). Such a cascade effect could influence the existing microbial community (Wurzburger and Wright 2015; Poirier et al. 2018; Luo et al. 2022; Kengdo et al. 2023) and, in the longer term, could even induce shifts in microbial community composition, which may further influence nutrient cycling, highlighting a critical aspect to be disentangled in future studies.

### Fine root dynamics with base cations addition

We predicted that adding base cations would elicit similar root responses compared to P addition, reducing biomass. However, we found no changes in root stock biomass with base cation addition. In most tropical experiments that manipulated single base cations, fine root stocks decreased, contrasting the findings in our study. For instance, adding K reduced fine root stocks after two years of fertilisation in a semi-deciduous tropical forest in Uganda (Manu et al. 2024) and in a lowland tropical rainforest in Panama (Yavitt et al. 2011). The difference between these results and ours may be due to the varying natural nutrient concentrations in the soils of each area. In Uganda and Panama, the average values of the sum of bases in the 0–10 cm soil layer show significant variation across regions: Panama shows values of 13.4 cmol_c_ kg^−1^ (Yavitt et al. 2011), Uganda reaches 43.1 cmol_c_ kg^−1^ (Manu et al. 2024), with our study site displaying low values of 0.43 cmol_c_ kg^−1^ (Ribeiro, 2023), comparable to other Ferralsols in the Amazon basin (Alvarez et al. 1996). Thus, our observed stability of root stock biomass across base cations additions and years may indicate adaptations to chronically low base cations availability with low plasticity of the root biomass to change.

We predicted that root turnover rates would increase with base cation addition. However, we did not find support for that in year 1, and contrary to expectations, fine root turnover decreased with base cations addition in the second year, extending root lifespan by nearly four months compared to plots without base cations addition. We also observed significant interactions between base cations and P during the second year, where the simultaneous addition of base cations and P (+ CAT + P) offsets the positive effect of P, bringing fine root turnover back to levels similar to the no base cations and no P condition (-CAT-P). The decrease in root turnover without changes in root stocks and root productivity (Lugli et al. 2021; Cunha et al. 2022) found in our study might suggest roots shifting towards more conservative mechanisms (McCormack and Iversen 2019; Bergmann et al. 2020). In fact, alleviation from base cations limitation may manifest by increasing root lifespan contrary to responses to P addition (Manu et al. 2024) because base cations are generally mobile in the soil solution (Epstein and Bloom 2006; Quesada et al. 2010), allowing easier root access without extensive investment in foraging. In contrast, the naturally low total P levels in our site (88 mg kg^−1^), combined with the low mobility of P in the soil (Cole and Heil 1981), may favour extensive root foraging for nutrient uptake when P availability is increased.

With increased cation availability, fine roots may acquire more nutrients per unit of root biomass through morphological and anatomical adaptations and possibly associations with mycorrhizal fungi without changes in root biomass (Weemstra et al. 2020). For instance, Lugli et al., (2021) found that after one year of the experiment in the same study areas in central Amazon, cation addition increased fine root diameter and arbuscular mycorrhizal (AM) colonisation. If these effects persisted into the second year of fertilisation, the increased mycorrhizal root colonisation could benefit trees in the cation treatment by enhancing soil foraging and nutrient uptake without changes in root stocks or productivity (Comas et al. 2014; Lugli et al. 2021). From a cost–benefit perspective, investments in arbuscular mycorrhizas might be less C-costly for trees and result in higher nutrient acquisition rates (sensu “outsourcing”) than investing in new fine roots that would explore the soil themselves (sensu “do-it-yourself”) (Bergmann et al.; 2020 and Lugli et al.; 2021). Therefore, future studies may determine whether the increased root lifespan observed in our experiment is linked to a possible increased investment in arbuscular mycorrhizal associations or alternative nutrient acquisition strategies.

Beyond their potential influence on mycorrhizal interactions, base cations play essential physiological roles in plants, contributing to structural integrity, enzymatic activation, and stress tolerance. Calcium (Ca) primarily supports cellular structure and long-distance internal transport, Mg acts as an activator of vital enzymes, while K regulates osmotic potential, supports enzymatic functions in respiration and photosynthesis, and enhances abiotic stress tolerance (Taiz and Zeiger 2013; Hasanuzzaman et al. 2018). Since the P treatment also contains high amounts of Ca and elicited a different effect than cation addition, we suggest that the observed response to base cations may be driven by K or Mg.

The differing soil mobility of base cations and P and their potential effects on root morphology and root symbiont associations may explain the contrasting outcomes in root lifespan. While two years of base cations addition may not have elicited strong responses on its own, it significantly influenced phosphorus' impact on roots. Thus, we can hypothesise that base cations may contribute to root longevity by enhancing structural stability and reducing environmental stress. Additionally, this dual function, both ecological and physiological, could improve nutrient use efficiency and extend root lifespan, counteracting the results we found for P addition. Although the mechanisms underlying these interactions remain complex, base cations are increasingly recognised as key regulators of tropical forest functioning, influencing not only root physiology but also broader ecosystem dynamics (Yavitt et al. 2011; Wurzburger and Wright 2015; Wright 2019; Bauters et al. 2022; Chen et al. 2024; Manu et al. 2024). We highlight the need for long-term investigation into the role of P and base cations in mediating root traits and plant–microbe interactions and their implications for root dynamics in tropical forests.

### Fine root dynamics with N addition

Most forests along the Amazon basin grow in ancient soils with high N availability, as N tends to accumulate during soil development through biological fixation and atmospheric deposition (Hedin et al. 2003). As predicted, no significant changes in fine root dynamics were expected with the addition of N, despite a potential trend towards reduced turnover in year 2. Therefore, adding N to an already N-rich system did not prompt any significant changes in fine roots, at least in the short term. However, the observed trend of reduced turnover in year 2 suggests that, over time, N addition may gradually influence root longevity. Although these effects were not statistically significant within our study period, they could become more pronounced with prolonged N enrichment. Furthermore, the significant interaction between N and base cations in year 2 suggests that these two nutrients added separately or in combination result in 30% lower root turnover rates compared to when neither N nor base cations are added. Such changes could have more pronounced long-term impacts on belowground C dynamics, directly by altering root traits and biomass or indirectly through mechanisms such as shifts in soil pH. Since base cations can help buffer the potential soil acidification caused by N addition, changes in pH may influence nutrient availability and soil microbial communities, affecting plant efficiency in resource acquisition and ecological interactions (Wright 2019; Luo et al. 2022). However, without further information on changes in soil properties and functional expression of root traits, we cannot fully interpret the ecological significance of such intricate interactions. Long-term studies are essential to determine whether this trend marks the onset of a more substantial shift in root turnover dynamics and what it could mean for C and nutrient cycling in this system.

## Conclusion

Over the two-year experiment, fine roots responded to increased availability of rock-derived nutrients, particularly P. The addition of P significantly accelerated fine root turnover, whereas cation addition extended root lifespan. Notably, the addition of base cations mitigated the effects of P, and, to a lesser extent, mediated the effects of N addition. These contrasting responses between P and base cations underscore their distinct soil mobility, roles in plant function, and contributions to nutrient uptake and utilisation strategies. Such shifts in fine root dynamics may have far-reaching implications for whole-plant C use efficiency and, consequently, for soil and ecosystem-wide C residence times over the long term. Our findings highlight that nutrient availability plays a crucial role in regulating fine root dynamics and C cycling, offering critical insights into how soil nutrient limitations could shape the response of Amazon forests to global environmental changes. It is worth noting that the observed responses may partly reflect short-term adjustments to an abrupt increase in nutrient availability, particularly at levels so much higher than seasonal flushes of nutrients common at the onset of the wet season in naturally nutrient-poor tropical forest soils. Long-term investigation is required to determine whether these changes persist or represent transient plasticity in root functions**.** These initial insights into belowground dynamics open numerous pathways for future research to investigate the influence of nutrient availability on morphological and physiological root traits, as well as plant-soil interactions.

## Supplementary Information

Below is the link to the electronic supplementary material.

ESM 1 DOCX (3.55 MB)

## Data Availability

The fine root productivity datasets analysed during the current study are available in the NERC Environmental Information Data Centre repository 10.5285/b3a55011-bf46-40f5-8850-86dc8bc4c85d. The fine roots biomass datasets analysed during the current study are available from the corresponding author on reasonable request.

## Abbreviations

AFEX: Amazon Fertilisation Experiment
BDFFP: Biological Dynamics of Forest Fragments Projec
Ca: Calcium
C: Carbon
LME: Linear mixed effects models
INPA: National Institute for Amazonian Research
NPP: Net primary productivity
N: Nitrogen
Mg: Magnesium
P: Phosphorus
K: Potassium
STRI: Smithsonian Tropical Research Institute
