# Supplementary material for: Phosphorus and base cations drive contrasting root dynamics in a central Amazon forest
Source: Plant Soil. 2026 Feb 2;520(1):957–72. doi: 10.1007/s11104-026-08303-2 (PMC13038795; doi:10.1007/s11104-026-08303-2)
Supplement: Supplementary file 1 — DOCX (3.55 MB) [file 11104_2026_8303_MOESM1_ESM.docx]

Supplementary information

**FULL TITLE: PHOSPHORUS AND BASE CATIONS DRIVE CONTRASTING ROOT DYNAMICS IN A CENTRAL AMAZON FOREST**

**Journal:** Plant and Soil **Authors:**

Jéssica Schmeisk do Vale Rosa ^1^ - ORCID: 0000-0001-8286-8521 ✉

Kelly M. Andersen ^2,3^ - ORCID: 0000-0002-1460-9904

Amanda L.Cordeiro ^4^  - ORCID: 0000-0001-7226-0133

Anna Carolina Martins Moraes^1^ - ORCID: [0000-0002-2541-5320](https://orcid.org/0000-0002-2541-5320)

Ana Cláudia Francisco Salomão ^5^

Rafael Leandro de Assis ^1,6^  - ORCID: 0000-0001-8468-6414

Raffaello Di Ponzio ^1,7^  - ORCID: 0000-0002-7527-3594

Renata Vilar de Almeida ^5^  - ORCID: 0000-0003-4634-5293

Maria Pires Martins ^1^ - ORCID: 0000-0001-8094-1879

Hellen Fernanda Viana Cunha ^1^  - ORCID: 0000-0001-9205-3895

Nathielly Pires Martins ^8^  - ORCID:[0000-0002-2501-747X](https://orcid.org/0000-0002-2501-747X)

Sheila Trierveiler de Souza ^1^ - ORCID:0000-0001-7988-8548

Gyovanni Augusto Alcides Ribeiro ^1^  - ORCID: 000-0003-2210-2713

José Augusto Salim ^9^ - ORCID: 0000-0002-8675-7068

Érick Oblitas ^1^  - ORCID: [0000-0001-9344-2411](https://orcid.org/0000-0001-9344-2411)

Sara Deambrozi Coelho ^1^

Adriana C. Conceição ^1^

Bruno Takeshi Tanaka Portela ^1^  - ORCID: 0000-0002-1223-6665

Oscar J. Valverde-Barrantes ^1,10^ - ORCID: 0000-0002-7327-7647

José Luís C. Camargo ^1,5^  - ORCID: 0000-0003-0370-9878

Patrick Meir ^11, 12^ - ORCID:0000-0002-2362-0398

Anja Ramming ^8^ - ORCID:0000-0001-5425-8718 12

Iain P. Hartley ^2^ - ORCID: 0000-0002-9183-6617

Carlos Alberto Nobre Quesada ^1^

Laynara F. Lugli ^1,8^  - ORCID: 0000-0001-8404-4841

**Affiliations:**

1

Coordination of Environmental Dynamics, National Institute for Amazonian Research, Manaus, Brazil.

2

Geography, Faculty of Environment, Science and Economy, University of Exeter, Exeter, UK.

3

Latin America Department, Missouri Botanical Garden, Saint Louis, MO, USA

4

Dept. of Plant & Microbial Biology, University of Minnesota, St Paul, MN, USA

5

Biological Dynamics of Forest Fragment Project, National Institute for Amazonian Research, Manaus, Brazil.

6

Vale Institute of Technology, Belém, Brazil.

7

Programa de Pós Graduação em Ecologia, Conservação e Manejo da Vida Silvestre, Universidade Federal de Minas Gerais, Belo Horizonte, Brazil

8

TUM School of Life Sciences, Technical University of Munich, Freising, Germany.

9

Plant Biology Department, Institute of Biology, State University of Campinas, São Paulo, Brazil.

10

International Centre of Tropical Biodiversity, Department of Biological Sciences, Florida International University, Miami, USA

11

School of Geosciences, University of Edinburgh, Edinburgh, UK.

12

Research School of Biology, Australian National University, Canberra, Australian Capital Territory, Australia.

**Corresponding Author:**

✉ Jéssica Schmeisk-Rosa, PhD candidate

E-mail: [jessica.schmeisk@gmail.com](mailto:jessica.schmeisk@gmail.com)

Instituto Nacional de Pesquisas da Amazonia

Manaus, Amazonas, Brazil

**Table S1** Fine root stock (Mg C ha^-1^), productivity (Mg C ha^-1^ yr^-1^), and turnover ( yr^-1^), shown separately for the 0–10 cm and 10–30 cm soil layers, in a *terra firme* forest in central Amazon, Brazil, during the period from 2017 (Year 1) and 2019 (Year 2), in a nutrient addition experiment. Means ± 1SE considering n=4 to Control, and n=16 without or with nutrients are presented. Significance levels indicate differences among nutrient addition treatments within each year and soil layer, as follows: *** for p < 0.001, ** for p < 0.01, and * for p < 0.05. Year 1 productivity values are from Lugli et al. (2021), and Year 2 productivity values are from Cunha et al. (2022).

|  |  | Year 1 | | | | Year 2 | | | |
| --- | --- | --- | --- | --- | --- | --- | --- | --- | --- |
| Treatments | Depth | Stock | Productivity | Turnover | Stock | | Productivity | Turnover |  |
|  |  | (Mg C ha^-1^) | (Mg C ha^-1^ yr^-1^) | (yr^-1^) | (Mg C ha^-1^) | | (Mg C ha^-1^ yr^-1^) | (yr^-1^) |  |
| Control | 0-10 cm | 0.96 ± 0.15 | 0.73 ± 0.06 | 0.77 ± 0.09 | 0.88 ± 0.15 | | 0.66 ± 0.05 | 0.82 ± 0.15 |  |
| -P | 0-10 cm | 1.02 ± 0.05 | 0.89 ± 0.07 | 0.91 ± 0.08 | 0.90 ± 0.05 | | 0.56 ± 0.04 | 0.64 ± 0.05 |  |
| +P | 0-10 cm | 1.00 ± 0.05 | 1.16 ± 0.09 * | 1.20 ± 0.09 * | 0.85 ± 0.04 | | 0.81 ± 0.06 *** | 0.97 ± 0.08 *** |  |
| - CATIONS | 0-10 cm | 0.97 ± 0.05 | 0.94 ± 0.07 | 1.01 ± 0.09 | 0.84 ± 0.04 | | 0.76 ± 0.07 | 0.94 ± 0.09 |  |
| + CATIONS | 0-10 cm | 1.04 ± 0.06 | 1.11 ± 0.09 | 1.09 ± 0.09 | 0.91 ± 0.05 | | 0.60 ± 0.04 * | 0.68 ± 0.05 ** |  |
| - N | 0-10 cm | 1.03 ± 0.04 | 1.07 ± 0.09 | 1.05 ± 0.08 | 0.91 ± 0.05 | | 0.74 ± 0.05 | 0.87 ± 0.08 |  |
| + N | 0-10 cm | 0.99 ± 0.06 | 0.98 ± 0.08 | 1.05 ± 0.10 | 0.85 ± 0.04 | | 0.62 ± 0.06 * | 0.74 ± 0.07 |  |
| Control | 10-30 cm | 0.41 ± 0.06 | 0.58 ± 0.10 | 1.38 ± 0.03 | 0.43 ± 0.06 | | 0.44 ± 0.04 | 1.11 ± 0.24 |  |
| -P | 10-30 cm | 0.40 ± 0.03 | 0.64 ± 0.04 | 1.75 ± 0.16 | 0.48 ± 0.03 | | 0.35 ± 0.03 | 0.79 ± 0.09 |  |
| +P | 10-30 cm | 0.41 ± 0.03 | 0.73 ± 0.07 | 1.89 ± 0.17 | 0.47 ± 0.03 | | 0.51 ± 0.05 * | 1.20 ± 0.17 * |  |
| - CATIONS | 10-30 cm | 0.42 ± 0.03 | 0.65 ± 0.04 | 1.65 ± 0.17 | 0.47 ± 0.03 | | 0.46 ± 0.05 | 1.09 ± 0.16 |  |
| + CATIONS | 10-30 cm | 0.39 ± 0.03 | 0.73 ± 0.07 | 1.99 ± 0.17 | 0.48 ± 0.03 | | 0.40 ± 0.04 | 0.91 ± 0.12 |  |
| - N | 10-30 cm | 0.41 ± 0.02 | 0.72 ± 0.06 | 1.80 ± 0.15 | 0.46 ± 0.03 | | 0.46 ± 0.04 | 1.11 ± 0.15 |  |
| + N | 10-30 cm | 0.40 ± 0.04 | 0.65 ± 0.07 | 1.84 ± 0.19 | 0.49 ± 0.03 | | 0.40 ± 0.05 | 0.89 ± 0.12 |  |

**Table S2** Fine root stock (Mg C ha^-1^), productivity (Mg C ha^-1^ yr^-1^), and turnover ( yr^-1^), referring to the 0–30 cm soil layer**,** in a *terra firme* forest in central Amazon, Brazil, during the period from 2017 (Year 1) and 2019 (Year 2), in a nutrient addition experiment. Means ± 1SE considering n=4 to Control, and n=16 without or with nutrients are presented. Significance levels indicate differences among nutrient addition treatments within each year and soil layer, as follows: *** for p < 0.001, ** for p < 0.01, and * for p < 0.05. Year 1 productivity values are from Lugli et al. (2021), and Year 2 productivity values are from Cunha et al. (2022).

|  |  | Year 1 | | | Year 2 | | | |  |
| --- | --- | --- | --- | --- | --- | --- | --- | --- | --- |
| Treatments | Depth | Stock | Productivity | Turnover | | Stock | Productivity | Turnover | |
|  |  | (Mg C ha^-1^) | (Mg C ha^-1^ yr^-1^) | (yr^-1^) | | (Mg C ha^-1^) | (Mg C ha^-1^ yr^-1^) | (yr^-1^) | |
| Control | 0-30 cm | 1.37 ± 0.09 | 1.31 ± 0.14 | 0.95 ± 0.08 | | 1.32 ± 0.21 | 1.11 ± 0.06 | 0.92 ± 0.18 | |
| -P | 0-30 cm | 1.42 ± 0.07 | 1.54 ± 0.09 | 1.12 ± 0.08 | | 1.38 ± 0.07 | 0.91 ± 0.06 | 0.69 ± 0.06 | |
| +P | 0-30 cm | 1.41 ± 0.07 | 1.89 ± 0.15 * | 1.38 ± 0.11 * | | 1.33 ± 0.06 | 1.32 ± 0.10 * | 1.02 ± 0.08 * | |
| - CATIONS | 0-30 cm | 1.39 ± 0.06 | 1.59 ± 0.11 | 1.19 ± 0.11 | | 1.32 ± 0.06 | 1.23 ± 0.11 | 0.97 ± 0.09 | |
| + CATIONS | 0-30 cm | 1.43 ± 0.08 | 1.84 ± 0.15 | 1.32 ± 0.10 | | 1.39 ± 0.06 | 1.01 ± 0.08 | 0.74 ± 0.06 * | |
| - N | 0-30 cm | 1.44 ± 0.05 | 1.79 ± 0.13 | 1.26 ± 0.09 | | 1.37 ± 0.07 | 1.21 ± 0.09 | 0.93 ± 0.09 | |
| + N | 0-30 cm | 1.38 ± 0.09 | 1.63 ± 0.13 | 1.25 ± 0.11 | | 1.34 ± 0.06 | 1.02 ± 0.10 | 0.78 ± 0.08 | |

**Table S3** ANOVA summary for fine root stock in the 0–30 cm soil layer in 2017 (Year 1), in a *terra firme* forest in central Amazonia, based on a factorial nutrient addition experiment (P, base cations and N). The table shows the sum of squares (Sum Sq), mean square (Mean Sq), numerator and denominator degrees of freedom (NumDF and DenDF), F-value, and p-value (Pr(>F)) for each main effect and interaction. Statistical significance was considered at p < 0.05.

| Stock 2017 - Year 1 | | | | | | |
| --- | --- | --- | --- | --- | --- | --- |
|  | Sum Sq | Mean Sq | NumDF | DenDF | F value | Pr(>F) |
| N | 0.12853 | 0.12853 | 1 | 21 | 0.3484 | 0.5613 |
| P | 0.00462 | 0.00462 | 1 | 21 | 0.0125 | 0.9120 |
| CATIONS | 0.05059 | 0.05059 | 1 | 21 | 0.1371 | 0.7148 |
| N:P | 0.12004 | 0.12004 | 1 | 21 | 0.3254 | 0.5744 |
| N:CATIONS | 0.15549 | 0.15549 | 1 | 21 | 0.4215 | 0.5232 |
| P:CATIONS | 101,555 | 101,555 | 1 | 21 | 27,531 | 0.1119 |
| N:P:CATIONS | 0.91825 | 0.91825 | 1 | 21 | 24,893 | 0.1296 |

**Table S4** ANOVA summary for fine root stock in the 0–30 cm soil layer in 2019 (Year 2), in a *terra firme* forest in central Amazonia, based on a factorial nutrient addition experiment (P, base cations and N). The table shows the sum of squares (Sum Sq), mean square (Mean Sq), numerator and denominator degrees of freedom (NumDF and DenDF), F-value, and *p*-value (Pr(>F)) for each main effect and interaction. Statistical significance was considered at p < 0.05.

| Stock 2019 - Year 2 | | | | | | |
| --- | --- | --- | --- | --- | --- | --- |
|  | Sum Sq | Mean Sq | NumDF | DenDF | F value | Pr(>F) |
| N | 0.02894 | 0.02894 | 1 | 24 | 0.0710 | 0.7922 |
| P | 0.10871 | 0.10871 | 1 | 24 | 0.2666 | 0.6103 |
| CATIONS | 0.24435 | 0.24435 | 1 | 24 | 0.5993 | 0.4464 |
| N:P | 0.01247 | 0.01247 | 1 | 24 | 0.0306 | 0.8626 |
| N:CATIONS | 0.51947 | 0.51947 | 1 | 24 | 12,740 | 0.2702 |
| P:CATIONS | 0.43251 | 0.43251 | 1 | 24 | 10,607 | 0.3133 |
| N:P:CATIONS | 0.08453 | 0.08453 | 1 | 24 | 0.2073 | 0.6530 |

**Table S5** ANOVA summary for fine root productivity in the 0–30 cm soil layer in 2017/2018 (Year 1), in a *terra firme* forest in central Amazonia, based on a factorial nutrient addition experiment (P, base cations and N). The table shows the sum of squares (Sum Sq), mean square (Mean Sq), numerator and denominator degrees of freedom (NumDF and DenDF), F-value, and *p*-value (Pr(>F)) for each main effect and interaction. Statistical significance was considered at p < 0.05. . Year 1 productivity values are from Lugli et al. (2021).

| Productivity - Year 1 | | | | | | |
| --- | --- | --- | --- | --- | --- | --- |
|  | Sum Sq | Mean Sq | NumDF | DenDF | F value | Pr(>F) |
| N | 1.06 | 1.06 | 1 | 24 | 0.84 | 0.370 |
| P | 5.31 | 5.31 | 1 | 24 | 4.18 | 0.052 |
| CATIONS | 2.57 | 2.57 | 1 | 24 | 2.03 | 0.167 |
| N:P | 0.08 | 0.08 | 1 | 24 | 0.06 | 0.805 |
| N:CATIONS | 2.44 | 2.44 | 1 | 24 | 1.93 | 0.178 |
| P:CATIONS | 0.12 | 0.12 | 1 | 24 | 0.09 | 0.762 |
| N:P:CATIONS | 0.02 | 0.02 | 1 | 24 | 0.02 | 0.892 |

**Table S6** ANOVA summary for fine root productivity (Mg C ha⁻¹ yr⁻¹) in the 0–30 cm soil layer in 2018/2019 (Year 2), in a *terra firme* forest in central Amazonia. The model was simplified through backward selection from a full factorial model including P, base cations and N addition treatments. The final model retained only P as a fixed effect. Degrees of freedom (Df), sum of squares (Sum Sq), mean square (Mean Sq), F-value, and *p*-value are shown. Significance levels are indicated as follows: *** for p < 0.001, ** for p < 0.01 and * for p < 0.05. . Year 2 productivity values are from Cunha et al. (2022).

| Productivity - Year 2 | | | | | |
| --- | --- | --- | --- | --- | --- |
|  | Df | Sum Sq | Mean Sq | F value | Pr(>F) |
| P | 1 | 1.32 | 1.32 | 11.4 | 0.0021 ** |
| Residuals | 30 | 3.48 | 0.116 |  |  |

**Table S7** ANOVA summary for fine root turnover (yr⁻¹) in the 0–30 cm soil layer in 2017/2018 (Year 1), in a *terra firme* forest in central Amazonia. The model was simplified through backward selection from a full factorial model including P, base cations and N addition treatments. The final model retained only P as a fixed effect. Degrees of freedom (Df), sum of squares (Sum Sq), mean square (Mean Sq), F-value, and *p*-value are shown. Significance levels are indicated as follows: *** for p < 0.001, ** for p < 0.01 and * for p < 0.05.

| Turnover - Year 1 | | | | | | |
| --- | --- | --- | --- | --- | --- | --- |
|  | Sum Sq | Mean Sq | NumDF | DenDF | F value | Pr(>F) |
| P | 0.545 | 0.545 | 1.000 | 27 | 4.29 | 0.048 * |

**Table S8** ANOVA summary for fine root turnover (yr⁻¹) in the 0–30 cm soil layer in 2018/2019 (Year 2), in a *terra firme* forest in central Amazonia. The model was simplified through backward selection from a full factorial model including P, base cations and N addition treatments. The final model retained only P as a fixed effect. Degrees of freedom (Df), sum of squares (Sum Sq), mean square (Mean Sq), F-value, and *p*-value are shown. Significance levels are indicated as follows: *** for p < 0.001, ** for p < 0.01, * for p < 0.05 and . for p < 0.1.

| Turnover - Year 2 | | | | | | |
| --- | --- | --- | --- | --- | --- | --- |
|  | Sum Sq | Mean Sq | NumDF | DenDF | F value | Pr(>F) |
| N | 0.18 | 0.18 | 1.000 | 26.000 | 3.35 | 0.0787 . |
| P | 0.887 | 0.887 | 1 | 26 | 16.52 | 0.0004 *** |
| CATIONS | 0.419 | 0.419 | 1 | 26 | 7.81 | 0.0096 ** |
| N:CATIONS | 0.353 | 0.353 | 1 | 26 | 6.58 | 0.0164 * |
| P:CATIONS | 0.261 | 0.261 | 1 | 26 | 4.87 | 0.0364 * |
